# The Potential Use of Cannabis in Tissue Fibrosis

**DOI:** 10.3389/fcell.2021.715380

**Published:** 2021-10-11

**Authors:** Nazar Pryimak, Mariia Zaiachuk, Olga Kovalchuk, Igor Kovalchuk

**Affiliations:** Department of Biological Sciences, University of Lethbridge, Lethbridge, AB, Canada

**Keywords:** fibrosis, anti-fibrotic, *Cannabis sativa*, cannabinoids, inflammation

## Abstract

Fibrosis is a condition characterized by thickening or/and scarring of various tissues. Fibrosis may develop in almost all tissues and organs, and it may be one of the leading causes of morbidity and mortality. It provokes excessive scarring that excels the usual wound healing response to trauma in numerous organs. Currently, very little can be done to prevent tissue fibrosis, and it is almost impossible to reverse it. Anti-inflammatory and immunosuppressive drugs are among the few treatments that may be efficient in preventing fibrosis. Numerous publications suggest that cannabinoids and extracts of *Cannabis sativa* have potent anti-inflammatory and anti-fibrogenic properties. In this review, we describe the types and mechanisms of fibrosis in various tissues and discuss various strategies for prevention and dealing with tissue fibrosis. We further introduce cannabinoids and their potential for the prevention and treatment of fibrosis, and therefore for extending healthy lifespan.

## Introduction

Fibrosis is a pathology associated with the replacement of parenchyma with connective tissue during the healing process. Fibrosis is defined as an excessive growth, stiffness, and sometimes scarring of different tissues or organs along with an imputed overaccumulation of extracellular matrix (ECM) components and collagen (Liu, 2011). Fibrotic illness is not well understood, it has a poor outcome and is mainly untreatable, all of which is compared to the terminal stage of cancer (Wernig et al., 2017). This condition is a lifelong pathological anomaly that may occur in various organs (Table 1), with a higher frequency in the skin, liver, heart, kidneys, and lungs.

**TABLE 1:** Main types of fibrosis.

Organ/tissue	Type of fibrosis	References
Skin	Hypertrophic scar Systemic sclerosis	Rabello et al. (2014) Allanore et al. (2015)
Heart	Cardiac fibrosis Hypertrophic cardiomyopathy Cardiac dysfunction Valvular disease Arrhythmia	Hinderer and Schenke-Layland (2019) Ho et al. (2010) Khan and Sheppard (2006)
Bone marrow	Myelofibrosis Myelodysplastic syndrome Chronic myelogenous leukemia	Zahr et al. (2016) Hinderer and Schenke-Layland (2019)
Liver	Cirrhosis Portal hypertension Hepatocellular carcinoma	Šmíd (2020)
Retroperitoneum	Retroperitoneal fibrosis	Vaglio and Maritati (2016)
Gut	Intestinal fibrosis Enteropathies Inflammatory bowel disease	Hinderer and Schenke-Layland (2019)
Joint	Arthrofibrosis	Usher et al. (2019)
Brain and nervous system	Glial scar Alzheimer	Hinderer and Schenke-Layland (2019)
Eye	Subretinal fibrosis Epiretinal fibrosis Vision loss	Friedlander (2007) Khaw et al. (2020)
Lung	Idiopathic pulmonary fibrosis Cystic fibrosis Pulmonary hypertension Thromboembolic disease	Barratt et al. (2018) Friedlander (2007) Smith et al. (2013) Farghaly and El-Abdin (2015)
Mediastinum	Mediastinal fibrosis	Rajput et al. (2000)
Pancreas	Pancreatic fibrosis Cystic fibrosis Chronic pancreatitis Duct obstruction	Klöppel et al. (2004) Madácsy et al. (2018) Klöppel (2007) Apte et al. (2011)
Kidney	Renal fibrosis Cystic fibrosis Nephrogenic systemic fibrosis Chronic kidney disease Renal anemia	Liu (2011) Yahiaoui et al. (2009) Waikhom and Taraphder (2011) Panizo et al. (2021) Xiao and Liu (2013)

Different types of fibrosis have been recognized based on anatomical location such as pulmonary [idiopathic pulmonary fibrosis (IPF), cystic fibrosis, emphysema], liver (cirrhosis, portal hypertension, hepatocellular carcinoma) or skin (keloids, systemic sclerosis). The most well-known and studied example of fibrosis is IPF. This condition is a lifelong, incurable illness targeting lungs. This disease usually affects middle-aged people and older adults and is characterized by a long-lasting cough along with difficulties in breathing of an unknown origin; besides IPF is very difficult to diagnose. Many IPF patients struggle with an acute worsening of breathing that is correlated with high mortality. The progression rate of this condition is very unpredictable. Some patients can deteriorate very quickly, while others may remain asymptomatic for many years. There is no generally approved treatment for this disease. The development of treatments is focused on fibroproliferation and fibrogenesis (Liu, 2011; Fujimoto et al., 2015; Hoyer et al., 2019). Because of insensitivity to pharmacological treatments, an average survival time is 3 years.

Epidemiological data on fibrosis in different organs is well documented in the literature. For example, an incidence of IPF varies between 0.6 and 17.4 per 100,000 population per year (Ley and Collard, 2013), two third of all patients were 60 years and above, and the highest prevalence was reported among patients of 80 years and above – 165.9 per 100,000 population (Raimundo et al., 2016). In Caucasians, cystic fibrosis occurs roughly in 1 in 3,000–4,000 births; and among other races, cystic fibrosis is less frequent, 1 in 4,000–10,000 in Latin Americans and 1 in 15,000–20,000 in African Americans, and even less in Asian Americans (Sanders and Fink, 2016). As to liver cirrhosis, according to 2017 data, 112 million compensated cases were reported worldwide (Sepanlou, 2020), and in patient who were more than 65 years old, a risk of severe liver fibrosis was 3.78 times higher (Kim et al., 2015). Also, more than 100 million cases of keloid are reported annually worldwide (Gauglitz et al., 2011; Li et al., 2017).

There is generally no good treatment of fibrosis and complete recovery is nearly impossible. Treatment includes various anti-inflammatory and anti-fibrotic agents with various degree of success. In this respect, cannabinoids and *Cannabis sativa* extracts, known for their strong anti-inflammatory potential, may be serve as useful additives to treatment of fibrosis. In this review, we will cover the pathology of fibrosis and current therapy of fibrosis as well as introduce basic components of endocannabinoid system (ECS) and propose potential use of cannabinoids for treatment of fibrosis.

## Phases of Normal Wound Healing and Fibrosis

In most cases, fibrosis occurs after acute or more often chronic damage to tissues, followed by abnormal repair. There are two ways of repair of the injured tissues. The first one is the regeneration by the propagation of undamaged cells of parenchyma and the maturation of stem cells – normal wound healing process. The second one is scar tissue formation through the accumulation of connective tissues – tissue fibrosis. The regeneration is a possibility of damaged tissues to be repaired and their defective elements to be restored. Cells that remain undamaged are able to proliferate and maintain the structure of the tissue. In some cases, fibrosis may occur due to a critical tissue injury or as a result of the inability of injured tissue to accomplish the repair. Fibrosis occurs due to either a large amount of collagen deposition associated with the long-lasting inflammation or ischemic necrosis. Cell proliferation is handled by growth factors, although the central role is played by ECM and maturation of stem cells (Occleston et al., 2010).

Different types of cells, such as fibroblasts, vascular endothelial cells, and some fragments of injured tissues proliferate along with the repair of damaged tissues. In fibrosis and scarring, tissue repair is characterized by the proliferation of connective tissues rather than parenchymal tissues that happens upon normal regeneration (Galliot et al., 2017).

### Phases of Wound Healing

**FIGURE 1:**
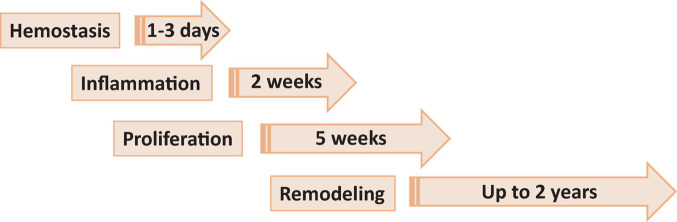
Phases of wound healing. Phase 1, hemostasis is the process of clot formation to stop bleeding, and includes steps such as vasoconstriction, aggregation of platelets and migration of leukocytes. Phase 2, inflammation is the process of cleaning the wound and preparing for the formation of new blood vessels. It includes processes such as release of antibacterial molecules by neutrophils, engulfing of pathogens and debris by macrophages, and release of angiogenic substances to stimulation angiogenesis and granulation. Phase 3, proliferation (or granulation) – the process allowing to bring the wound edges together and seal it. It includes proliferation of the wound by fibroblasts, with secretion of glycoproteins and collagen, followed by migration of epithelial cells from the wound edges and formulation of granulation tissues. Phase 4, remodeling (or maturation) phase is mostly a continuation of proliferation phase resulting in formation of proper tissue.

Wound healing consists of four main phases, including hemostasis, inflammation, proliferation or granulation and remodeling or maturation, each phase lasting from days to months (Figure 1).

Abnormal wound healing resulting in scar formation also includes similar phases/steps, such as inflammation, cell proliferation, and remodeling, but is characterized by more extensive deposition of collagen, fibrin, fibronectin, etc. (Profyris et al., 2012).

#### The First Phase – Hemostasis

**FIGURE 2:**
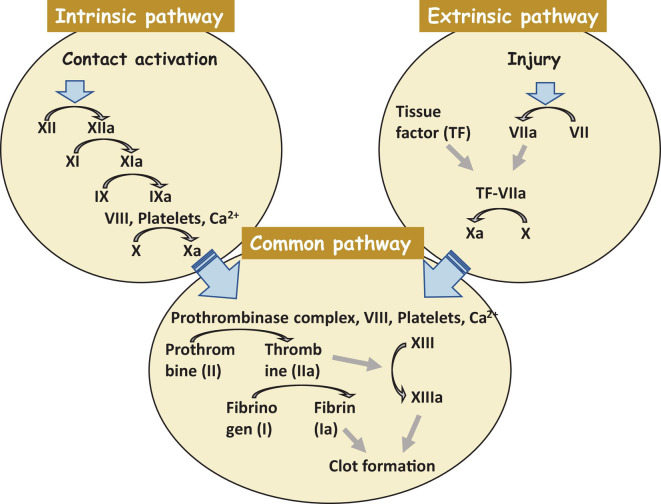
A clot formation cascade. There are three steps of the clotting (coagulation) cascade: the intrinsic pathway (factors XII, XI, IX, and VIII), the extrinsic pathway (factor VII), and the common pathway. During clotting, cascade factor X may be activated by the extrinsic and intrinsic pathways. The common pathway consist of steps from the activation of factor X to the clot formation. Factors that are activated are shown with a lowercase “a”.

The most crucial step is not to restore a tissue but to stop bleeding from the injured place. Coagulation starts exactly after trauma and finishes within hours. Collagen assists this process in the damaged area. Hemostasis consists of two subphases, *primary* and *secondary* hemostasis. Primary hemostasis is the formation of a plug at the injured place where endothelial cells become exposed. In the secondary hemostasis, there are two main pathways of blood clotting: the *extrinsic* and the *intrinsic* pathways, and they come together in the common pathway. The extrinsic pathway is a primary stage in plasma mediated secondary hemostasis. Due to tissue damage, tissue factor (TF also known as platelet tissue factor or factor III) is released in the plasma, which results in binding of factor VIIa and calcium to boost the activation of factor X to Xa (Figure 2). The intrinsic pathway includes factors I (fibrinogen), II (prothrombin), IX (Christmas factor), X (Stuart–Prower factor), XI (Plasma thromboplastin), and XII (Hageman factor) (Robertson and Miller, 2018). The common pathway includes steps from the activation of factor X to the formation of active thrombin which brakes fibrin into a cross-linked complex.

#### The Second Phase – Inflammation

Inflammation plays a central role in normal wound healing and fibrosis. Tissue repair and regeneration also depend on the extent of injury and inflammation. When the injury is extensive in the presence of chronic inflammation, repair may predominate even when the damaged cells can regenerate.

In normal circumstances, the inflammatory microenvironment quickly handles the damaged particles or pathogens. The essential factors of inflammation and fibrogenesis are summarized below (Table 2; Newton and Dixit, 2012; Kendall and Feghali-Bostwick, 2014).

**TABLE 2:** Key mediators of inflammation and fibrogenesis.

	Substance	Production site	Effects	References
Profibrotic factors acting on fibroblasts	TGFβ	White blood cells	Transformation of resident (subcutaneous, pulmonary, etc.) fibroblasts to myofibroblasts. Stimulation of collagen and fibronectin transcription. Stimulation of resting monocytes and inhibition of activated macrophages.	Frangogiannis (2020)
	IL-1β	Fibroblasts, macrophages	Inflammation promotion and fibrotic responses (in part, through activation of TNFα).	Lopez-Castejon and Brough (2011)
	IL-6	T cells, skeletal muscle cells, macrophages	Regulation of inflammation (pro- and anti-inflammatory). Stimulation of cellular differentiation and fibrosis.	Tanaka et al. (2014)
	IL-13	Mast cells, T lymphocytes, eosinophils and basophils	Stimulation of TGFβ production, proliferation of fibroblasts, collagen and MMP production.	Marone et al. (2019)
	IL-33	Smooth muscle cells, epithelial and endothelial cells	Signals through ST2 to initiate and enhances profibrogenic cytokine production in a macrophage-dependent manner.	Li et al. (2014)
	TNFα	Macrophages, T lymphocytes, NK cells, mast cells, eosinophils	Stimulation of inflammation and fibrosis, in part through TGF-β signaling pathway, activation of myofibroblasts and increased secretion of MMPs.	Yoshimatsu et al. (2020)
	FGFs	Various parenchymal cells	Fibrosis enhancement through binding and activation of fibroblast growth factor receptor (FGFR).	Xie et al. (2020)
	PDGF	Platelets, smooth muscle cells, endothelial cells and macrophages	Stimulation differentiation, proliferation, and ECM production via interaction with PDGFα and PDGFβ receptors on myofibroblasts.	Klinkhammer et al. (2018)
	Leukotrienes (LTB4, LTC4, LTD4, LTE4)	White blood cells	Stimulation of fibroblasts proliferation and production of the matrix via modulation of the production of cyclic AMP by interaction with G-protein adenylate cyclase.	Kowal-Bielecka et al. (2007)
Profibrotic factors released from fibroblasts	VEGF	Macrophages, fibroblasts, platelets	Angiogenesis promotion. Facilitates monocyte recruitment and infiltration of fibrotic tissues mediated through a VEGF-dependent sinusoidal permeability, leading either to resolution or promotion of fibrosis.	Yang et al. (2014)
	IL-1	Fibroblasts	Facilitates inflammation and fibrosis through autocrine stimulation of IL-1 receptor.	Kelly et al. (2019)
	IL-6	Fibroblasts	Facilitation of inflammation and fibrosis through binding of IL-6 to IL-6Rα receptor, which then associates with the signal-transducing gp130 protein to facilitate phosphorylation of the transcription factor STAT-3. Phosphorylated STAT-3 regulates expression of pro-fibrotic genes.	Kobayashi et al. (2015)
	IL-33	Dermal and cardiac fibroblasts	Promotion of inflammation and fibrosis by signaling through ST2 and activating TGFβ production.	Kotsiou et al. (2018)
	Angiotensin II	Macrophages and myofibroblasts	Promotion of TGFβ mediated heart remodeling. Fibrosis enhancement via the angiotensin type 1 receptor (AT1).	Rosenkranz (2004)
	IGFII	Fibroblasts	Stimulation of fibrosis through mannose-6-phosphate/insulin-like growth factor receptor (M6P/IGFII receptor) in turn activating latent transforming growth factor β (L-TGF-β).	Ghahary et al. (2000)
	IGFBP-3		Fibrosis initiation and enhancement by binding IGF-I and ECM components, inducing the production of extracellular matrix components such as collagen type I and fibronectin. Inhibit IGF mediated proliferation (via MEK/ERK and PI3K/AKT).	Pilewski et al. (2005)
	IGFBP-5			Yasuoka et al. (2009)
Antifibrotic factors acting on fibroblasts	PGE_2_	Almost all nucleated cells	Inhibition of fibroblast proliferation and suppression of collagen production. Promotion of normal fibroblast apoptosis through EP2/EP4 signaling and a reduction in the Akt activity.	Huang et al. (2009)
	HGF	Fibroblast	Prevents fibrosis and induces tissue repair acting through Met receptor and supporting the growth in epithelial and endothelial cells, but not in myofibroblasts.	Panganiban and Day (2011)
	PPAR ligands	Expressed in almost all tissues	Potent antifibrotic effects, reduction of β-catenin levels. Regulate the fate determination of mesenchymal cell lineage.	Jeon et al. (2017) Vallée et al. (2017)

#### The Third Phase – Proliferation and Granulation

Cell proliferation is an essential component of tissue repair, wound healing and fibrogenesis. There are several types of cells, such as epithelial cells, endothelial cells, and fibroblasts that participate in fibrogenesis and normal process of healing of the wound.

Mesothelial cells originate from the embryonic mesoderm and play an essential role during trauma or infection. For instance, in pleural injuries, they assist in transporting white cells. Also, as a result of mesothelial-to-mesenchymal transition (MMT), these cells, might be genetically reprogrammed after the influence of specific stimuli. In a recent mouse model, the lineage analysis of stem cells demonstrated that MMT increased the proliferation of myofibroblasts and hepatic satellite cells during liver fibrogenesis (Tirado and Koss, 2018).

Fibrocytes are of a mesenchymal origin and are phenotypically inactive due to a low amount of rough endoplasmic reticulum. These cells produce fibroblastic components such as collagen, fibronectin, and vimentin. When influenced by TGF-β, they can produce alpha-smooth muscle actin (α-SMA) which plays a role in angiogenesis and immunity. Fibrocytes can also migrate to the damaged area with blood flow (de Oliveira and Wilson, 2020).

Fibroblasts originate from the embryonic mesoderm tissues. Due to the chemotaxis feature, fibroblasts are able to migrate within tissue in response to chemical stimuli. In case of injury, they can cause contraction of the matrix that leads to the sealing of the open wound. Fibroblasts play an important role in fibrogenesis, for example, TGF-β1 dependent differentiation into myofibroblasts (Weiskirchen et al., 2019).

Epithelial cells are located in different areas of the body, such as skin, urinary tract, blood vessels, and internal organs. One of the critical features is their ability to differentiate into different types of cells. During epithelial-to-mesenchymal transition (EMT), epithelial cells become transited cells that become sensitive to the fibroblast’s specific protein (FSP1). The plasticity of epithelial cells allows them to become a source of myofibroblasts in the damaged cells (Macara et al., 2014).

Endothelial cells are mainly responsible for the formation of a barrier in the endothelium of capillaries, venules, vein, arterioles, and arteries. Being stimulated by TGF-β, endothelial cells can release α-SMA and become able to convert into mesenchymal cells (endothelial-to-mesenchymal-transition, EndMT). It was demonstrated that EndMT could lead to fibrosis in the organs such as heart, kidney, and lungs (Sakai and Tager, 2013).

Pericytes are fibroblast-like cells that surround endothelial cells in blood vessels. Pericytes are able to contract and consequently control blood flow. In the case study, it was suggested that this type of cells produce α–SMA, neural/glial antigen (NG2) and platelet-derived growth factor receptor-β (PDGFR-β). Moreover, they are a source of myofibroblasts in pulmonary tissues. Another study reported that Foxd1 progenitor-derived pericytes prominently lead to the lung fibrosis (Sakai and Tager, 2013).

Vascular smooth muscle cells are responsible for the relaxation and contraction of blood vessels. As a result of the injury, they produce α–SMA, vimentin, desmin, and other compounds. It has also been shown that collagen type I is induced by bradykinin secretion in vascular smooth muscle cells through the TGF-β1 activation (Liu et al., 2017).

**FIGURE 3:**
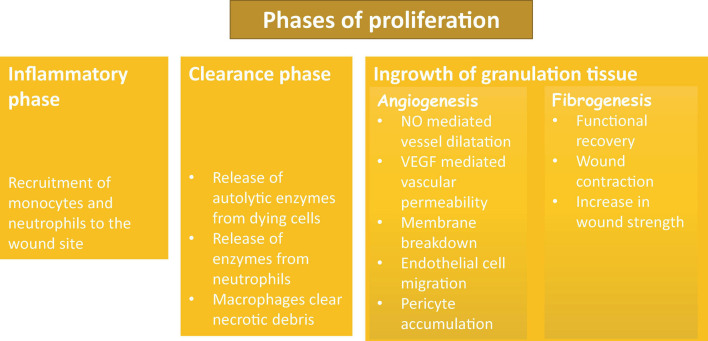
Phases of proliferation and granulation.

There are two main processes involved in proliferation phase of repair: *formation of granulation tissues* and *wound contraction*. Wound contraction usually starts on day 2–3 and is finished within 2 weeks. The primary cells that are responsible for this process are myofibroblasts, the unique cells that have features of fibroblasts and smooth muscle. The main role of these cells is the contraction of the wound by up to 80%. Granulation tissue is soft in touch and has a pink color. Granulation is a sign of tissue repair; it is formed by three steps: the inflammatory phase, the clearance phase, and the ingrowth of granulation tissue (Figure 3). During the *inflammation phase*, cells that are predominantly involved in the process are monocytes and neutrophils. The *clearance phase* is characterized by the release of autolytic enzymes from dying cells as well as enzymes from neutrophils; macrophages also clear necrotic debris. The final phase is the *ingrowth of granulation tissue* during which granulation tissue is formed. This phase can be divided into two processes: angiogenesis and fibrogenesis (Baum and Duffy, 2011; Bochaton-Piallat et al., 2016; Alhajj et al., 2020).

Angiogenesis (neovascularization) is the development of blood vessels. Angiogenesis could be the result of sprouting either from pre-existing blood vessels or from stem cells. There are a few steps in the angiogenesis from pre-existing blood vessels. The first one involves *vessel dilation* that is mediated by NO, and the second step includes an *increased vascular permeability* that is mediated by the vascular endothelial growth factor (VEGF). The next step is a *breakdown of the basement membrane* and the formation of a vessel sprout. The other step is the *migration of endothelial cells* toward chemotactic and *angiogenic stimuli* that cause a proliferation of endothelial cells and their maturation leading to capillary tube remodeling. The final phase of angiogenesis is the *accumulation of periendothelial cells* (pericyte) (Papetti and Herman, 2002).

Angiogenesis from stem cells develops from endothelial precursor cells (EPC) stored in the bone marrow, and if needed, they migrate to the place of injury (Aldair and Montani, 2010).

#### The Fourth Phase – Healthy Remodeling or Remodeling With Fibrogenesis

**FIGURE 4:**
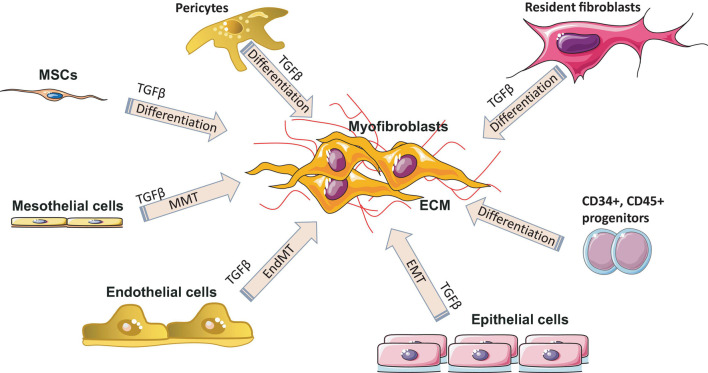
Myofibroblast origin in fibrosis. Resident fibroblasts, pericytes, circulating progenitor cells (CD34^+^, CD45^+^, bone marrow–derived mesenchymal stem cells (MSC) transition, mesothelial cells undergoing mesothelial-to-mesenchymal transition (MMT), epithelial cells undergoing epithelial to mesenchymal transition (EMT) and endothelial cells undergoing endothelial-mesenchymal transdifferentiation (EndMT) are all known sources of myofibroblasts in various fibrotic diseases. ECM, extracellular matrix; TGF-β, transforming growth factor-β.

Remodeling (maturation phase) after injury usually takes place from several weeks to months or years and depends on what type of tissue is damaged, injury location, and the associated comorbidities (infections, arteriosclerosis, vein thrombosis, nutritional status, diabetes, and some drugs). During remodeling phase, rate of synthesis of collagen by fibroblasts exceeds the rate at which it is degraded, resulting in continuous increase in the amount of collagen. Remodeling includes three steps: *functional recovery*, *wound contraction* and an *increased tensile strength* of the wound (Cañedo-Dorantes and Cañedo-Ayala, 2019). The maturation phase is characterized by the formation of scar tissue as well as by the absence of inflammatory cells (neutrophils, macrophages) and the termination of blood vessel proliferation. Granulation tissue in the scar is replaced by dense collagen. The scar initially consists of a provisional matrix that contains fibrin, fibronectin, and collagen type III, but later on, collagen type III is replaced by collagen type I (Reinke and Sorg, 2012). The next step is wound contraction, with the main goal being a reduction of a gap between two cut margins. Myofibroblasts play a key role during this phase. Figure 4 shows all major processes of differentiation, activation or transition of various cells into myofibroblasts. Collagen type I is responsible for the last step – an increase in the strength of the wound. The recovery of ∼80% of the original tissue strength will usually take up to 3 months.

Skin wound healing can be subdivided into *primary* and *secondary unions* (Alhajj et al., 2020). By primary union (first intention), regeneration occurs with a minimum scaring tissue, for example, a clean surgical wound. By secondary union (secondary intention), the wound has the larger tissue defects with a wide distance between edges; wound healing by secondary intention occurs by regeneration and scarring. In some cases, due to abnormal wound healing, keloids or hypertrophic scars might occur. In a hypertrophic scar, there is a build-up of extra collagen fibers, which results in the elevation of the scar. Fibrillar collagen fibers are located parallel to the epidermis with a lumpy red scar, and they do not extend beyond the original scarring area. Usually, hypertrophic scars affect younger individuals with the delayed healing of wounds caused by underlying conditions such as infections, and usually, there is an improvement with the treatment. Morphologically keloids are characterized as eosinophilic, focally fragmented complexes of haphazardly arranged collagen. Also, in comparison with hypertrophic scars, one-third of keloids have α–SMA- expressing myofibroblasts. The scar tissue in keloids grows beyond the inflammation area, and it is difficult to treat (Moshref et al., 2010).

### Physiological Injury Healing vs. Pathological Fibrosis

Fibrosis of the organ tissues is caused by parenchymal cell destruction (*alteration* or *injury* phase); as a result of tissue trauma, macrophages become active and enter the damaged area. Also, local immune cells create chemokines and cytokines which activate mesenchymal cells located close to the injury area. The next step is the initiation of the production of ECM and the elevated manufacturing of pro-inflammatory cytokines and angiogenic factors (Weiskirchen et al., 2019). After trauma, cells produce inflammatory mediators that provoke the anti-fibrinolytic coagulation cascade, the first step of which is the coagulation. During this stage, known as *inflammation* stage, platelets are activated and form fibrin clots. Next, platelets liberate inflammatory chemokines. Then the infiltration of leukocytes happens into the injured site, and they excrete profibrotic cytokines (TGF-β and IL-13). Neutrophils are typically engaged in the infiltration process earlier than lymphocytes and macrophages (Rosales, 2018).

The *proliferation* stage follows the inflammation stage; during this stage, fibroblasts become active, and myofibroblasts induce and deposit ECM that will be a framework through the tissue regeneration action. The last step is *remodeling* (Gonzalez et al., 2016). In physiological recovery, the extra volume of ECM is degraded, myofibroblasts and fibroblasts go through apoptosis, and inflammatory cells leave the recovered tissues. On the other hand, the fibrosis process extends inflammation, and myofibroblasts stimulate the elevated accumulation of ECM which leads to the creation of a perpetual fibrotic scar. The contrasting features that distinguish fibrosis from normal wound healing are chronic inflammation, the persistence of myofibroblast activity, MMP-TIMP imbalance, and the excessive ECM deposition. These differences are very important to be understood from the therapeutic point of view because drugs can be prescribed to target these particular molecular disturbances.

Fibroblasts control synthesis and catabolism of collagen as well as an increase in collagen amount by MMPs and their inhibitors (tissue inhibitors of TIMPs). Changing the balance between these mechanisms will cause the elevation or dropping of collagen amount inside the injured area. In addition, an increasing number of mesenchymal cells will aggravate response. During the remodeling phase, fibroblasts synthesize collagen at a higher rate than they degrade it, leading to the continuous accumulation of collagen. Generally, inflammation stimulates fibrosis. According to some reports, however, fibrosis is not always driven by inflammation. This fact clarifies the shortage of efficacy of anti-inflammatory mediators in the management of the fibrotic disease (Wynn, 2007; Kryczka and Boncela, 2015).

### Fibrosis Prevention and Treatment Options

Prevention strategies for the development of fibrosis are very important in the modern world because life expectancy and the patients’ quality of life are expected to rise gradually. When patients are aware of avoidable risk factors for this condition, they, for example, should quit smoking to prevent the development of pulmonary fibrosis and should treat all acute diseases in time to prevent the development of chronic conditions. There are unavoidable factors such as genetics, the existing comorbidities (diabetes mellitus, herpes virus infection), the environmental exposures, air pollution as well as chronic use of some medications that also have to be considered (Zaman and Lee, 2018).

Anti-inflammatory drugs are widely used to manage fibrosis due to a strong connection between inflammation and fibrosis (Suthahar et al., 2017; Lands and Stanojevic, 2019; Simon et al., 2019). As a result of better understanding of the pathology of fibrosis, molecular targets of this condition and modern drugs affecting it have been recently discovered. A single-component medication is characterized by the presence of a single component that can target either extracellular or intracellular factors. The main extracellular targets are MMPs, growth factors, TNF. Most of the drugs targeting intracellular factors are small molecules; they can easily translocate inside the cytoplasm compared to other large molecules, like monoclonal antibodies.

There are four categories of intracellular factors that can be targeted by anti-fibrotic medicine: nuclear receptors, enzymes, other proteins, and epigenetic factors (Li et al., 2017). The antifibrotic medications suppress kinases located in the cytoplasm, and moreover, they inhibit the translocation of transcription factors responsible for the expression of profibrotic genes.

Epigenetic regulators represent a very specific category of anti-fibrotic treatment. The main targets of epigenetic-based management of fibrosis are microRNAs (miRNAs). Anti-miRs – are miRNA oligonucleotides that are able to complementary bind to miRNAs involved in fibrosis and neutralize them when deposited inside the cell. miRNAs like let-7, miR-21, miR-29, miR-155 play an important role in fibrosis, particularly in TGF-β control. Let-7 and miR-29 are antifibrotic; in contrast, miR-21 and miR-155 are profibrotic, and their expression will rise during the fibrosis. On the other hand, the decreased expression of miR-29 in systemic sclerosis (SSc) fibroblasts leads to the increased levels of type I and III collagen. The reduction of miR-29 was noted in the fibrotic reaction in the lungs, heart, and kidneys. IL-4, TGF-β, and PDGF-B reduced the level of miR-29 in SSc fibroblasts as well as in the bleomycin-induced model of skin fibrosis (Maurer et al., 2010; Harmanci et al., 2017). According to another study, miR-21 was highly elevated in animal and human models of transplant kidney nephropathy. MiR-21^–/–^ mice experienced less interstitial fibrosis in response to kidney injury; this was pheno-copied in wild-type mice that were treated with anti-miR-21 oligonucleotides. The peroxisome proliferator-activated receptors (Pparα) and Mpv17l are two main metabolic pathways that are key targets for miR-21. Also, miR-21 down-regulated inhibitors of angiogenesis and migration, especially the RECK (the reversion-inducing cysteine-rich protein with Kazal motifs) and the atypical matrix metalloproteinase (MMP) inhibitor that led to the enhanced MMP activity in kidney injury (Chau et al., 2012). As a result of the administration of oligonucleotides that silenced miR-21, a reversal of the deleterious action of miR-21 in kidney injury was noted. Some studies demonstrated a significant effect of miR-21 on pulmonary and cardiac fibrosis (Thum et al., 2008).

In multi-component therapy, several approaches are combined, with numerous ingredients acting on numerous targets. In fibrosis, there are multiple pathological pathways and multi-component drugs that are able to modulate these pathways and create synergistic effects. In the Table 3, single and multi-component medications used nowadays in the treatment of fibrosis are summarized (Li et al., 2017).

**TABLE 3:** Single- and multi-component medications targeting fibrosis factors.

Group	Target or mechanism type	Mechanism of action	Drug name	Disease	Reference/trial identifier
**Single-component medications targeting extracellular factors**					
Growth factor	Extracellular TGF-β signaling	TGF-β, inhibitor	Pirfenidone	IPF	Margaritopoulos et al. (2016)
	PDGF/VEGF	PDGFR, antagonist	Imatinib	SSc, nephrogenic systemic fibrosis, IPF	NCT00677092 NCT00613171 NCT00131274
		VEGFR/PDGFR, antagonist	Nintedanib	Scleroderma, IPF	NCT02597933 NCT01335464
	TNF	TNF, inhibitor	Talidomide	IPF	NCT00162760
		TNF, inhibitor	Etanercept	IPF	NCT00063869
		TNF, inhibitor	Belimumab	SSc	NCT01670565
Cytokines	Interleukin	IL-1R1, antagonist	Anakinra	Cystic fibrosis	Iannitti et al. (2016)
		IL-1 βR, antagonist	Rilonacept	SSc	NCT01538719
	Interferon	IFN-γR, stimulant	Actimmune	IPF, cystic fibrosis, liver fibrosis	NCT00047658 NCT00043303 NCT00043316
MMP/TIMP	MMP/TIMP	MMP/TIMP, inhibitor	Marimastat	Liver fibrosis	de Meijer et al. (2010)
Other proteins and peptides	Endothelin	ET-1 receptor, antagonist	Macitentan	IPF	NCT00903331
			Bosentan	IPF	NCT00070590 NCT00319696 NCT01395732
			Ambrisentan	IPF, SSc	NCT00879229 NCT01051960
	Angiotensin II	AT1 receptor, antagonist	Losartan	Cystic fibrosis, Liver fibrosis	NCT00298714 NCT03206788
	GPCR	Prostacyclin receptor, agonist	Iloprost	SSc	NCT00109681
			Treprostinil	IPF, SSc	NCT00703339 NCT00775463
**Single-component medications targeting intracellular factors**					
Enzymes	mTOR	mTORC1/2, inhibitor	Rapamycin (Sirolimus)	Renal intestinal fibrosis	NCT01079143
	JAK-STAT	JAK1/JAK2, inhibitor	Ruxolitinib	Myelofibrosis	NCT00952289
	PI3K-Akt	Akt, inhibitor	Omipalisib	IPF	NCT01725139
	MAPK	MAPK, inhibitor	MMI-0100	IPF, cardiac fibrosis	Xu et al. (2014)
	NF-kB	IKK, inhibitor	IMD-1041	Cardiac fibrosis	Tanaka et al. (2012)
Nuclear receptors	PPAR	PPAR-γ, agonist	Rosiglitazone	Liver fibrosis	NCT00492700
Other proteins	Intracellular TGF-β signaling	SMAD2/3, inhibitor	Pirfenidone	IPF, SSc	NCT00287729 NCT01933334
		SMAD3/4, inhibitor	Pentoxifylline	Skin fibrosis	NCT00001437
		SMAD3, inhibitor	SiS-3 Glycyrrhizin	Renal fibrosis, liver fibrosis	NCT00686881 Meng et al. (2015)
Epigenetics	miRNA	miR-21, inhibitor	Anti-miR-21	IPF, renal fibrosis	Liu et al. (2010) Chau et al. (2012)
**Multi-component drugs**					
	TGF-pc/MMP-2c		Fuzhenghuayu capsule (FZHY)	Liver fibrosis	NCT00854087 NCT02241616
	TNF-α/TGF-β		Danggui-Buxue-Tang (DBTG)	IPF	Lv et al. (2012)

*TGF-β, transforming growth factor-β; PDGF, platelet-derived growth factor; PDGFR, platelet-derived growth factor receptor; VEGF, vascular endothelial growth factor; VEGFR, vascular endothelial growth factor receptor; TNF, tumor necrosis factor; IFN-γR, interferon-γ receptor; MMP, matrix metalloproteinase; TIMP, tissue inhibitor of metalloproteinase; ET-1 receptor, endothelin-1 receptor; AT1 receptor, angiotensin II receptor type 1; GPCR, G protein-coupled receptor; mTOR, mechanistic target of rapamycin; mTORC1, mechanistic target of rapamycin complex 1; JAK-STAT, janus kinase/signal transducers and activators of transcriptions; PI3K-Akt, phosphoinositide 3-kinase/protein kinase B; MAPK, mitogen-activated protein kinase; NF-kB, nuclear factor kappa-light –chain- enhancer of activated B cells; IKK, I-kappa B kinase; SMAD3, mothers against decapentaplegic homolog 3.*

Only nintedanib and pirfenidone have been approved by FDA for the treatment of fibrosis, particularly of IPF, and ruxolitinib has been approved by FDA for the treatment of myelofibrosis; other medications are still experimental, and some of them are undergoing clinical trials the results of which might help improve the understanding of the fibrosis pathway.

Nintedanib medication is a small molecule kinase inhibitor that reduces the proliferation and migration of lung fibroblasts. Nintedanib inhibits receptor tyrosine kinases (RTKs), for instance, FGFR1-3, VEGFR1-3, Fns-like tyrosine kinase-3 (FLT3), PDGFR α and β. Also, this drug inhibits kinase signaling pathways. Its main side effects are nausea, diarrhea, and liver dysfunction (Wind et al., 2019; Valenzuela et al., 2020). In clinical practice, a long-term use of nintedanib is still discussed. Research in this area will help improve patient outcomes (Valenzuela et al., 2020). Pirfenidone treatment mainly reduces fibroblast proliferation and causes the inhibition of collagen synthesis and down-regulation of profibrotic cytokines. As a result of inhibition, it causes the suppression of TGF-β2 mRNA levels and TGF-β2 protein and the suppressed expression of the TGF-β pro-protein convertase furin. Also, this drug decreases the MMP-11 protein levels. It should be noted that pirfenidone has some severe side effects such as photosensitivity, nausea, stomach pain and many others that may lead to medication intolerance and discontinuation (Hughes et al., 2016; Margaritopoulos et al., 2016; Moran-Mendoza et al., 2019). Ruxolitinib is widely used in myelofibrosis treatment. This drug is a kinase inhibitor that is selective for JAK1 and 2. The main role of these kinases is the regulation of growth factor signaling and cytokine release. The known side effects of this medicine are anemia, thrombocytopenia, increased liver enzymes, and diarrhea (Elli et al., 2019).

### The Role of Cannabinoids and Cannabis in Inflammation and Fibrosis

Recently, the ECS has received a significant attention from mainstream medical professionals, being viewed as an important therapeutic target for many pathological conditions. Human physiology significantly depends on a proper function of this system. The ECS has been established as an important homeostatic regulator. It affects almost all functions of the body. It consists of endocannabinoids (2-AG, AEA), their metabolic enzymes and receptors, including cannabinoid receptors 1 (CB1), cannabinoid 2 (CB2), transient receptor potential channels of the vanilloid subtype 1 and 2 (TRPV1, TRPV2), G protein-coupled receptors 18, 55, 119 (GPR18, GPR55, GPR119) (Laezza et al., 2020). Cannabinoid receptors are highly expressed in cells involved in two subtypes of immunity, adaptive and innate. For example, CB1 and CB2 receptors are expressed in the natural killer cells, macrophages, T and B cells (Chiurchiù, 2016). In general, activation of the CB2 receptor leads to anti-inflammatory effects. Since the expression of CB1 receptors is lower in the immune cells than the expression of CB2 receptors, their function in the immune system still remains controversial (Turcotte et al., 2016). CB2^–/–^ mice exhibit abnormalities in the development of T and B cells (Ziring et al., 2006). Cannabinoids, mainly by modulating the expression of ECS receptors induce apoptosis of immune cells, and inhibit their proliferation; suppress pro-inflammatory cytokines production, and induce T-regulatory cells (Nagarkatti et al., 2009). The imbalance in the ECS can significantly impact the proper functioning of the whole organism, including fibrosis and inflammation processes. For example, the activation of the CB1 receptor leads to fibrogenesis, while the enhancement of the CB2 receptor inhibits fibrosis progression (Mallat et al., 2011). In animal models, it was demonstrated that the deletion of CB1 caused an improvement of liver fibrosis, whereas CB2 deletion resulted in an elevated amount of collagen accumulation and an increased inflammation (Patsenker and Stickel, 2016). Concerning inflammation, the use of CB2 receptor agonists was documented to inhibit the infiltration of inflammatory cells into liver tissue. In addition, CB2 receptor knockout mice had the more profound inflammation and damage to the liver than wild-type mice (Batkai et al., 2007).

Cannabis is widely known as a plant with psychoactive properties. It includes over 500 compounds such as different cannabinoids, terpenes, terpenoids, fatty acids, and flavonoids. Cannabinoids (known as phytocannabinoids in contrast to endocannabinoids) act via the ECS. The most abundant are cannabidiol (CBD) and Δ9-tetrahydrocannabinol (Δ9-THC); they are the most studied cannabinoids with numerous documented medicinal properties (Zurier and Burstein, 2016; Lafaye et al., 2017). The uniqueness of the effect of cannabis extracts is in entourage effect. Often, but not always, cannabis extracts have more profound effects on various disease and conditions than isolated cannabinoids (Kovalchuk and Kovalchuk, 2020). This is due to modifying effects of minor cannabinoids, terpenes and other molecules that frequently act as amplifiers, acting on the same receptors. Looking at the enormous varieties of cannabis cultivars nowadays, it is clear that research in this field should continue to discover new possibilities of fibrosis treatment (Russo, 2019).

#### Cannabinoids as Anti-inflammatory Agents

According to previous reports, some of the cannabinoids can be used as anti-inflammatory agents (Zurier and Burstein, 2016; Wang et al., 2020). Currently in medical practice, these substances have little documented negative effect on patients in comparison with other drugs. Cannabinoids have other mechanisms of action on inflammation in comparison with nonsteroidal anti-inflammatory drugs (NSAIDs). NSAIDs inhibit the activity of cyclooxygenase enzymes, prostaglandins (Zurier and Burstein, 2016). Recently, it has been discovered that cannabinoid receptor signaling regulates the proliferation and function of fibroblasts which are crucial cells in scar formation (Nagarkatti et al., 2009). By suppressing inflammation, they may stop the progression of repair by scarring. It has been shown that cannabinoids and cannabis extracts suppress the pro-inflammatory cytokines IL-2, IL-1β, TNF-α, IFN-γ, IL-12, IL-8, IL-6 in different cell lines and animal models. Due to this, the inflammatory process will be prominently inhibited (Nagarkatti et al., 2009).

One of the anti-inflammatory mechanisms of cannabinoids is through the regulation of mitochondrial homeostasis. CBD has been shown to alleviate cerebral ischemia in rats by reducing brain oedema, blood-brain barrier permeability, infarction size, and neurological deficit. This effect was due to the increased expression of Na^+^/Ca^2+^ exchanger proteins (Khaksar and Bigdeli, 2017). When blood flow is restored in the ischemic area, it causes inflammation and oxidative-stress-related injury in the affected area. CBD has demonstrated a neuroprotective effect in oxygen–glucose-deprivation/reperfusion *in vitro* model by reducing the oxidative stress, improving mitochondrial bioenergetics and modulating the glucose metabolism (Sun et al., 2017). In contrast, THC treatment of the trophoblast cell line, HTR8/SVneo, showed a reduction in mitochondrial respiratory function and membrane potential. This data suggested that THC can cause dysfunction of mitochondria (Walker et al., 2021). When THC effect was evaluated on mitochondria, extracted from the rat brain, similar results were obtained: it enhanced oxidative stress and induced mitochondrial dysfunction in the brain (Wolff et al., 2015).

Cannabis extracts were even proposed to be used for prevention and treatment of COVID-19. Significant inhibitory effects on the key receptor protein Ace2 required for SARS-Cov2 virus entry in the gateway entry tissues was found in response to several cannabis extracts (Wang et al., 2020). Similarly, several other extracts were found to be potent inhibitors of major pro-inflammatory molecules responsible for severe COVID-19 progression (Wang et al., 2020).

It was also shown that cannabis users living with HIV have lower neuroinflammation. This was confirmed by demonstrating that marijuana users had lower levels of CD16+ monocytes and inducible protein 10 (IP-10) compared to HIV-infected patients non-cannabis users (Rizzo et al., 2018).

#### Cannabinoids as Anti-fibrotic Agents

A lot of research has already been done, and currently many studies are undergoing on the use of endo-, synthetic, and phytocannabinoids in the fibrosis field. In one *in vivo* study where a mouse model of type I cardiomyopathy was used, it was demonstrated that CBD treatment diminished the diabetes-associated cardiac fibrosis. A significant decrease of collagen deposition and the expression of profibrotic genes like MMP-2, MMP-9, TGF-β, connective tissue growth factor, fibronectin andcollagen-1 were noted (Montecucco and Di Marzo, 2012).

Liver fibrosis is a usual complication of many long-lasting liver illnesses such as viral hepatitis B and C, non-alcoholic steatohepatitis, drug-induced liver injury, alcohol abuse, and autoimmune conditions. In long-lasting liver damage, the activated hepatic stellate cells (HSCs) and myofibroblasts are the main contributors to the development of liver cirrhosis and hepatocellular cancer (Fu et al., 2011). An *in vitro* study performed on HSCs documented that CBD induced the programmed cell death of these cells (Lim et al., 2011). This effect was independent of cannabinoid receptors and was the result of endoplasmic reticulum stress induction. In addition, CBD enhanced the pro-apoptotic pathway IRE1/ASK1/c-Jun N-terminal kinase, which resulted in HSCs death. This CBD-induced programmed cell death of activated HSCs was confirmed *in vitro* in human, mouse and rat cell lines, but not in the quiescent cell lines. The well-known fact that the activated HSCs play a crucial role in the development and continuation of liver fibrosis supports the fact that cannabis extracts might be turned into promising antifibrotic drugs as they lead to the selective apoptosis of activated HSCs. The results of this study are very encouraging for further investigation of CBD *in vivo* (Lim et al., 2011). In addition, a meta-analysis of nine studies performed on 5,976,026 patients concluded that marijuana did not elevate the prevalence or progression of liver fibrosis in patients with hepatitis C or hepatitis C HIV co-infection (Hosein Mohimani et al., 2017). Also, it was noted that marijuana users had a reduced prevalence of non-alcoholic fatty liver disease (NAFLD). Furthermore, these patients consumed more sodas and alcohol, therefore the healthy lifestyle was not a cause of the reduced prevalence of NAFLD. This effect might be induced by reducing fat depositions via omega-3 fatty acids and the impact of CBD on insulin sensitivity (Hosein Mohimani et al., 2017).

Concerning the effect of THC, it has been shown that it also inhibits the proliferation of liver myofibroblasts and stellate cells via CB2 receptors and leads to their programmed cell death. Due to this, THC may also possess antifibrotic properties (Tam et al., 2011).

The endocannabinoid AEA also demonstrated the anti-fibrogenic features by suppressing the proliferation of HSC and induction of necrosis. The elevated AEA levels were documented in cirrhotic patients, which might be a response to fibrosis. This endogenous cannabinoid can trigger the topical inflammatory response and systemic dilatation of vessels, therefore the opportunity for fibrosis treatment was restricted (Parfieniuk and Flisiak, 2008). Another endocannabinoid, 2-AG, was considered as a fibrogenic agent. When used in higher doses *in vitro* on HSC, it activated fibrosis via the membrane cholesterol-dependent mechanism (Tam et al., 2011). Another endogenous cannabinoid, oleoylethanolamide (OEA), was used in a mouse model of hepatic fibrosis and showed the inhibition of collagen deposition and suppression of collagen type I and III gene expression, α-SMA, MMP2, MMP9, and TIMP1. These effects were mediated through the PPARα mechanisms (McVicker and Bennett, 2017).

Synthetic cannabinoids were also shown to be beneficial for fibrosis treatment. An *in vitro* study performed on pulmonary fibroblasts demonstrated that JWH133, a CB2 receptor agonist, suppressed the collagen type I and α-SMA and inhibited the proliferation and migration of fibroblasts. These effects were reversed by the use of a CB2 receptor antagonist, SR144528. *In vivo* studies on bleomycin-induced lung fibrosis in mice, showed that JWH133 decreased the lung density, and the fibrotic score and histological results illustrated the suppression of the collagen accumulation and inflammatory response. In both models, this particular synthetic cannabinoid inhibited the crucial pathway of fibrogenesis, TGF-β1/Smad2 (Fu et al., 2017). WIN-55,212, a nonselective CB1 and CB2 receptor agonist as well as JWH133 were assessed on the mouse model of systemic sclerosis. They prevented the development of dermal and pulmonary fibrosis and inhibited the proliferation of fibroblasts. CB2^–/–^ mice developed a significantly enhanced skin and lung fibrosis compared with CB2^+/+^ mice, indicating significant influence of the CB2 receptor on fibrosis development (Servettaz et al., 2010). Rimonabant, a CB1 receptor antagonist, was assessed on rat models of liver cirrhosis induced by carbon tetrachloride. Fibrosis was prominently suppressed by the use of this synthetic cannabinoid in rats compared with rats in the vehicle group. Rimonabant downregulated the fibrogenic (TIMP-1, TGF-β, MMP13, MMP2, MMP9, MMP1, MMP8) and inflammatory mediator (TNF-α, MCP-1) genes. In addition, Rimonabant treatment induced a prominent increase in the expression of the CB2 receptor (Giannone et al., 2012). Another study demonstrated that chronic stimulation of CB2 receptor with selective CB2 receptor agonist, JWH-133, leads to regression of fibrosis in cirrhotic rats. This selective agonist suppressed the inflammatory infiltrate, decreased fibrosis, lowered the number of activated hepatic stellate cells, and improved arterial pressure in comparison to the vehicle group. In addition, JWH-133 reduced levels of α-SMA and collagen and elevated levels of MMP-2 in the liver tissue of rats with cirrhosis in comparison with untreated rats with cirrhosis. This data provided promising results for the possibility to use selective CB2 receptor agonists as a treatment modality of hepatic fibrosis in humans (Muñoz-Luque et al., 2008). Another study tested the effect of a selective CB2 receptor agonist, AM1241, on myocardial fibrosis post-myocardial infarction in mice. The echocardiography results demonstrated that AM1241 significantly enhanced cardiac function; downregulated expression of collagen I, collagen III, TIMP-1, and plasminogen activator inhibitor (PAI)-1. When primary cardiac fibroblasts were exposed to hypoxia and serum deprivation to simulate ischemia, AM1241 was able to reduce α-SMA, collagen I and collagen III; this effect was partially abrogated by the Nrf2 siRNA transfection. Moreover, the CB2 receptor agonist, AM1241, activated and enhanced the translocation of Nrf2 to the nucleus and inhibited the TGF-β1/SMAd3 pathway. These data suggest that activation of the CB2 receptor might be one of the key targets to combat heart fibrosis after myocardial infarction (Li et al., 2016). The chronic peripheral pharmaceutical blockage of CB1 receptor (by SLV319 or JD5037 selective CB1 receptor antagonists) or genetic inactivation of CB1 receptors in the renal proximal tubule cells reduced kidney inflammation, suppressed tubulointerstitial fibrosis, and diminished diabetic-induced changes in the kidneys in mice. Also, the downregulation of the CB1 receptor suppressed glucose transporter 2, which resulted in reduced glucose reabsorption. These data supported the fact that peripheral CB1 receptor antagonists might be useful in treating patients with diabetic nephropathy (Hinden et al., 2018). A synthetic analog of THC, ajulemic acid (lenabasum), being a CB2 agonist, significantly prevented the development of bleomycin-induced skin fibrosis in mice and suppressed its progression. In addition, it inhibited collagen synthesis by skin fibroblasts obtained from patients with scleroderma. These effects were achieved by stimulating PPAR-γ signaling (Gonzalez et al., 2012). Lenabasum has been shown to have an anti-inflammatory capacity by directly affecting both arachidonic acid pathways (Burstein, 2021). Lenabasum was the only synthetic cannabinoid studied in human fibrotic diseases. A Phase II, randomized, placebo-controlled trial in adults suffering from systemic sclerosis showed promising results (NCT03398837); a high improvement rate on the immunosuppressant therapy background, and high improvement on mycophenolate that has an anti-fibrotic activity and prevents pulmonary deterioration in pulmonary fibrosis was observed. Unfortunately, the primary endpoint was not met during this trial (Spiera et al., 2021). Table 3 summarizes type of cannabinoids and their mechanism of action (Table 4).

**TABLE 4:** Anti-fibrotic effect of cannabinoids.

Compound	The mechanism of action	References
**Endocannabinoids**		
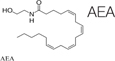	Suppressing the proliferation of HSCs and induces their necrosis	Parfieniuk and Flisiak (2008)
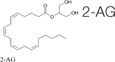	Generally considered as a fibrogenic agent, however, it is able to suppress fibrosis via the membrane cholesterol-dependent mechanism.	Tam et al. (2011)
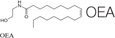	The inhibition of collagen deposition and suppression of collagen type I and III gene expression, α-SMA, MMP2, MMP9, and TIMP1. These effects were mediated through the PPARα mechanisms.	McVicker and Bennett (2017)
**Phytocannabinoids**		
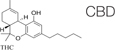	The apoptosis induction of HSCs as result of the induction of endoplasmic reticulum stress and the enhancement of the pro-apoptotic pathway IRE1/ASK1/c-Jun N-terminal kinase.	Lim et al. (2011)
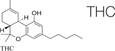	The inhibition of miofibroblast proliferation and stellate cells, the induction of their apoptosis via CB2 receptors.	Tam et al. (2011)
**Synthetic cannabinoids**		
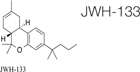	The suppression of collagen type I and α-SMA, inhibition of fibroblast proliferation and migration. The down-regulation of the TGF-β1/Smad2 pathway.	Fu et al. (2017) Muñoz-Luque et al. (2008)
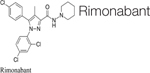	The suppression of expression of fibrogenic mediators (TIMP-1, TGF-β, MMP13, MMP2, MMP9, MMP1, MMP8, TNF-α, MCP-1)	Giannone et al. (2012)
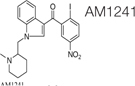	The downregulation of expression of collagen I, collagen III, TIMP-1, and plasminogen activator inhibitor. The inhibition of the TGF-β1/SMAd3 pathway.	Li et al. (2016)
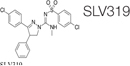	The suppression of glucose transporter 2; reduction in glucose reabsorption.	Hinden et al. (2018)
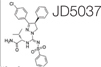	The suppression of glucose transporter 2; reduction in glucose reabsorption.	Hinden et al. (2018)
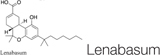	Stimulating PPAR-γ signaling	Gonzalez et al. (2012)

### Into Potential Mechanisms of Anti-fibrotic Effects of Cannabinoids – Role of MicroRNAs

**FIGURE 5:**
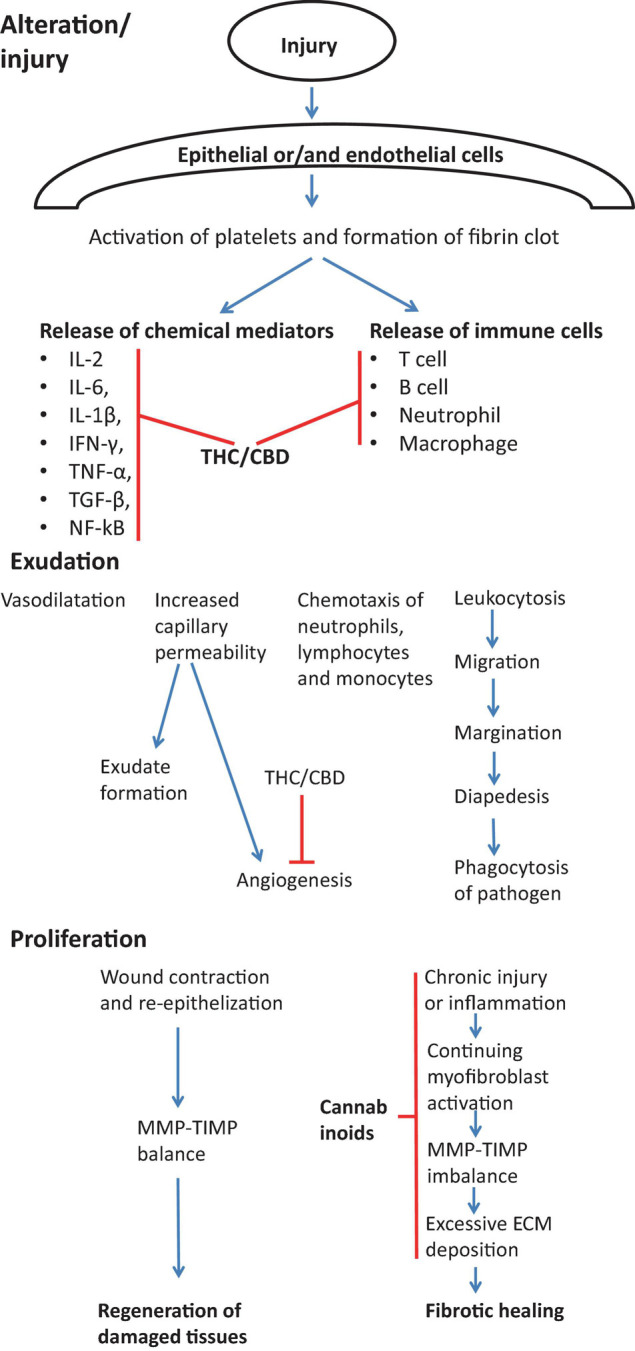
Effect of cannabinoids on various stages of wound healing in view of preventing/treating fibrosis. During the first stage of wound healing cannabinoids are responsible for inhibiting immune response by suppressing the release of immune cells into the injury cite and by inhibiting Th1 response and main pro-inflammatory cytokines (IL-2, IL-6, IL-1β, IFN-γ, TNF-α, TGF-β, NF-kB) (Watzl et al., 1991; Srivastava et al., 1998; Dinu et al., 2020). During exudation stage, cannabinoids inhibit the angiogenesis (Wietecha and DiPietro, 2013). Cannabinoids also actively contribute to the proliferation stage by suppressing chronic inflammation (Costa et al., 2007; Gallily et al., 2018), myofibroblast activation (Garcia-Gonzalez et al., 2009), the excessive deposition of ECM components (Garcia-Gonzalez et al., 2009) and normalization of TIMP-MMP imbalance (Garcia-Gonzalez et al., 2009).

The mechanisms of anti-fibrotic effects of cannabinoids are likely very diverse. Cannabinoids may target all steps of normal and abnormal wound healing, as shown on Figure 5.

Recent reports demonstrate the role of miRNAs in regulation of cannabinoid receptors and anti-fibrotic effect of cannabinoids. miRNAs like miR-30b-5p, miR-21, miR-155, miR-146a, miR-141, and miR-222 appear to be pro-inflammatory, while miRNAs like miR-187, miR-149, miR-145, and miR-99b are anti-inflammatory.

miR-30b-5p was found to be involved in CB1-mediated NLRP3 inflammasome activation. The study performed on mice with liver injury induced by carbon tetrachloride (CCl4) or methionine-choline-deficient, and high fat (MCDHF) diet showed that there is a relationship between CB1 receptor and Nod-like receptor family pyrin domain containing 3 (NLRP3) inflammasome in liver inflammation. The expression of the CB1 receptor was increased in hepatic tissue of mice with liver injury. CB1 receptor agonist, arachiodonyl-2′-chloroethylamide, enhanced expression of NLRP3 inflammasome and its activation in macrophages. AM281, a CB1 receptor antagonist, inhibited NLRP3 expression and activation of the inflammasome and reduced hepatic inflammation in CCl4- and MCDHF-treated mice. When miR-30b-5p agomir was administered, it targeted NLRP3 and mitigated liver inflammation. These results suggested that CB1/miR-30b-5p axis is able to modulate the activation of NLRP3 inflammasome and expression of NLRP3 in macrophages in liver inflammatory disease (Yang et al., 2020).

Cannabinoids can alter the expression of miR-155, a master regulator of inflammation (Mahesh and Biswas, 2019). Administration of selective CB2 receptor agonist AM1241 significantly reduced expression of TLR4, α-SMA, TGF-β1, miR-155, p65 NFkB, TNF-α, IL-6, IL-1β and vimentin genes caused by thioacetamide. Moreover, it significantly upregulated E-cadherin, glutathione content, and superoxide dismutase. This study showed that AM1241 reduces fibrosis, by activating the CB2 receptor, and inhibiting TLR4/miR-155/NFκB p65 pathway (Ali et al., 2021).

Chronic administration of THC significantly increased the expression of anti-inflammatory miRNAs including miR-187, miR-149, miR-145, miR-99b, miR-24, and miR-10a dysregulated by acute infection with Simian Immunodeficiency Virus (SIV). THC administration also reduced viral load, gastrointestinal inflammation, and the overall disease progression (Chandra et al., 2015). Another study, performed on rhesus macaques with chronic SIV infection with an intention to cause fibrosis, showed that THC prevented fibrosis and upregulated ten miRNAs, including pro-inflammatory miRNAs miR-21, miR-141 and miR-222 and miR-204, that directly targets matrix metalloproteinase 8 (MMP-8), an enzyme that degrades collagen (Kumar et al., 2019). In addition, THC inhibited proliferation and activation of T cells, suppressed PD-1 expression, and elevated the percentage of anti-inflammatory macrophages in intestinal tissue. These results suggest that THC is an important modulator of miRNA expression and can suppress intestinal inflammation and may prevent lymph node fibrosis (Kumar et al., 2019).

In another study, lipopolysaccharide (LPS) was used to stimulate the BV-2 microglial cells, and CBD had a much more significant effect than THC on the expression of cluster miRNAs (Juknat et al., 2019). While LPS increased the expression of many pro-inflammatory miRNAs, including miR-155, miR-146a, and miR-21, associated with Toll-like receptor (TLR) and NF-κB signaling, the CBD suppressed the expression of miR-155 and miR-146a. Moreover, it was demonstrated that LPS modulates the Notch signaling pathway by increasing the mRNA expression of Notch ligand Dll1. CBD and THC were able to reduce the expression of this ligand. Since the CBD+LPS group increased the expression of miR-34a and downregulated the expression of its target gene Dll1, it was suggested that miR-34a could be involved in the Notch signaling pathway modulation and, as a result, in reducing inflammation (Juknat et al., 2019).

Cannabidiol was demonstrated to trigger apoptosis in human neuroblastoma cell lines by downregulation of let-7a expression and, as a consequence, upregulation of caspase-3 and several growth arrest genes. In addition, CBD upregulated the expression of has-mir-1972 and caused decreased expression of BCL2L1 and SIRT2 genes (Alharris et al., 2019).

Yet another study showed that THC treatment can suppress the activation of Th1/Th17 lineage commitment via regulation of miRNA expression (Sido et al., 2016). When C57BL/6 mice with delayed-type hypersensitivity were treated with THC, oedema and immune cells infiltration subsided at the place of antigen rechallenge. Delayed-type hypersensitivity caused an increased expression of miR-21 and inhibition of miR-29b; miR-21elevates the Th17 differentiation via inhibiting SMAD7, while miR-29b is IFN-γ inhibitor. THC was able to reverse this miRNA imbalance. Moreover, when primary cells from these mice were transfected with miR-21 inhibitor or miR-29b mimic, the expression of SMAD7 increased, and the expression of IFN-γ decreased (Sido et al., 2016).

In another study, when C3H/Hej mice were administered staphylococcal enterotoxin B (SEB), it caused an acute mortality; in contrast, mice that received THC had a 100% survival rate and did not show any respiratory distress signs, hunched posture, or fur ruffling. In addition, the THC administration significantly reduced the vascular leak in comparison to the staphylococcal enterotoxin B exposure group. The staphylococcal infection caused the induction of miRNA-17-92 cluster, including miR-18a, which targeted the inhibitor of PI3K/Akt pathway, leading to suppression of T regulatory cells (Rao et al., 2015).

Another study showed that THC administered in post-staphylococcal enterotoxin B exposure protected mice from acute respiratory distress syndrome and toxicity. THC significantly downregulated let7a-5p and miR-34-5p, which target SOCS1, FoxP3, NOS1, and CSF1R as well as inhibited the pro-inflammatory cytokines, such as TNF-α and IFN-γ. This study suggested that THC can alter miRNA expression in the lungs, suppress the cytokine storm, and as a consequence, might cause mitigation of SEB-mediated pulmonary injury (Mohammed et al., 2020).

A combination of THC plus CBD suppressed neuroinflammation in murine experimental autoimmune encephalomyelitis (EAE) model and suppressed Th1 and Th17 cells via modulating miRNA expression. In addition, this combinational treatment reduced levels of CD4+ T cells and pro-inflammatory molecules such as TNF-α, IL-1β, IL-6, IFN-γ, IL-17, and TBX21) while elevating anti-inflammatory molecules (IL-4, IL-10, TGF-β, STAT5b, and FoxP3). Microarray analysis of miRNA of CD4+ T cells showed that THC+CBD administration significantly inhibited miR-122-5p, miR-27b-5p, miR-155-5p, miR-150-5p, miR-146a-5p, miR-31-5p, miR-21a-5p and upregulated miR-7116 and miR-706-5p (Al-Ghezi et al., 2019).

miR-29a appears to be one of the regulators of response to cannabinoids. miR-29a diminishes diabetic nephropathy via modulation of CB1 signaling. Upregulated expression of the CB1 receptor, TNF-α, IL-6, IL-1β, collagen IV, and downregulated expression of PPAR-γ was noted in streptozotocin-induced diabetic mice. In contrast, overexpression of miR-29a in mice negatively regulated CB1 receptor, blocking upregulation of pro-inflammatory and fibrogenic compounds, and substantially decreasing kidney hypertrophy. The overexpression of miR-29a also renewed PPAR-γ signaling. These data demonstrated that interaction among miR-29a, CB1 receptor, and PPAR-γ signaling plays a significant role in protecting renal tissue from developing fibrosis (Tung et al., 2019).

### Cannabis and Cannabinoids May Replace the Known Therapies for Fibrosis

Due to the lack of effective therapies for fibrosis, new more effective and modern therapies with less side effects need to be developed. The currently used drugs suppress the fibrogenetic pathways and reduce the progression of fibrosis. Similarly, cannabis extracts can also affect key profibrotic factors and pathways. Cannabinoid signaling regulates the proliferation and function of fibroblasts which are crucial cells in scar formation. The active suppression of fibroblast proliferation leads to the inhibition of collagen formation and deposition. As previously explained, cannabinoids can actively suppress inflammation by downregulating the pro-inflammatory cytokines such as IL-2, IL-1β, TNF-α, IFN-γ, IL-12, IL-8, IL-6, IL-15 (Wang et al., 2020). Due to this effect of cannabis, the fibrosis progression may stop. In comparison with drugs currently applied for treating pulmonary fibrosis, cannabinoids can also suppress the MMP/TIMP, PPAR and other pathways involved in fibrogenesis (Table 5). We can conclude that cannabis affects the same pathways as other drugs currently used medicine in fibrosis treatment, but it has a little- documented negative effects on patients as compared to other drugs used.

**TABLE 5:** A comparison of target molecules in treatment of fibrosis using modern therapy vs. cannabis treatment.

A single-component medication:	Cannabis
**The mechanism of action of asingle-component medication vs. cannabis**	
• TGF-β inhibitor (Pirfenidone) • PDGFR antagonist (Imatinib) • TNF inhibitor (Talidomide, Etanercept, Belimumab) • IL-1 antagonist (Anakinra, Rilonacept) • IFN-γ stimulant (Actimmune) • MMP/TIMP inhibitor (Marimastat) • mTOR inhibitor (Rapamycin) • JAK-STAT inhibitor (Ruxolitinib) • PI3K-Akt inhibitor (Omipalisib) • MAPK inhibitor (MMI-0100) • NF-kB inhibitor (IMD-1041) • miR-21 inhibitor (Anti-miR-21)	• TGF-β inhibitor • TNF inhibitor • MMP/TIMP inhibitor

**The mechanism of action of a multi-component medication vs. cannabis**	

**A multi-component medication:**	**Cannabis**

• TGF-pc/MMP-2c inhibitor (Fuzhenghuayu) • TNF-α/TGF-β inhibitor (Danggui-Buxue-Tang)	• TGF-β1/Smad2 inhibitor

## Conclusion

Fibrosis is a pathological process that may affect many organs. Significant improvement in understanding the tissue fibrosis pathways may give us a chance in the future to discover an effective antifibrotic treatment. Many studies have been performed to understand the molecular mechanisms, the cellular basis, and the most prominent characteristics of fibrosis in human organs. Molecules like TNFα play a key role in the establishment of inflammation and pathogenesis of fibrosis. At the same time, TNFα may be used a therapeutic agent that can resolve the established pulmonary fibrosis (Redente et al., 2014), further confirming that we do not have a clear picture of mechanisms and pathways of fibrosis.

In most tissues and organs, the fibrosis mechanisms are similar, but the regeneration and regression processes are different across organs and tissues. Mainly this diversity is due to the difference in the regenerative capacity of each tissue or organ (Friedman, 2015).

Based on reports presented in this review, we propose that single cannabinoids and components of cannabis extracts can positively interact with the key profibrotic factors and pathways. In comparison with modern antifibrotic medications, cannabinoids have fewer negative effects on patient’s health.

We conclude that modulation of ECS should be a modern approach for the treatment of different fibrotic conditions. This aspect of treatment has not been sufficiently studied. More detailed research should be done to find a patient-oriented treatment and improve patients’ quality of life. It would be very encouraging to find the curative option for this devastating condition that will help millions of patients worldwide.

## Author Contributions

NP, OK, and IK contributed to the conceptualization and design. NP and MZ contribute to the draft preparation. OK and IK contributed to the analysis, editing, and supervision. All authors were involved in a review preparation and editing.

## Conflict of Interest

The authors declare that the research was conducted in the absence of any commercial or financial relationships that could be construed as a potential conflict of interest.

## Publisher’s Note

All claims expressed in this article are solely those of the authors and do not necessarily represent those of their affiliated organizations, or those of the publisher, the editors and the reviewers. Any product that may be evaluated in this article, or claim that may be made by its manufacturer, is not guaranteed or endorsed by the publisher.
